# Adverse events associated with hepatic arterial infusion chemotherapy and its combination therapies in hepatocellular carcinoma: a systematic review

**DOI:** 10.3389/fimmu.2025.1531249

**Published:** 2025-03-03

**Authors:** Ying Wu, Zhenpeng Zeng, Shuanggang Chen, Danyang Zhou, Gangling Tong, Duanming Du

**Affiliations:** ^1^ Department of Interventional Therapy, Shenzhen Second People’s Hospital, The First Affiliated Hospital of Shenzhen University, Shenzhen, China; ^2^ Department of Minimally Invasive Interventional Therapy, Sun Yat-sen University Cancer Center, Guangzhou, China; ^3^ StateKey Laboratory of Oncology in South China, Collaborative Innovation Center of Cancer Medicine, Sun Yat-sen University, Guangzhou, China; ^4^ Department of Oncology, Peking University Shenzhen Hospital, Shenzhen, China

**Keywords:** hepatic arterial infusion chemotherapy, hepatocellular carcinoma, adverse events, combination therapy, safety management

## Abstract

**Background:**

Hepatic arterial infusion chemotherapy (HAIC) has emerged as a promising treatment for unresectable hepatocellular carcinoma (HCC). However, the safety profiles of HAIC and its various combination therapies remain to be systematically evaluated.

**Methods:**

We systematically searched PubMed, Embase, Cochrane Library, and Web of Science databases from inception to November 2024. Studies reporting adverse events (AEs) of HAIC monotherapy or combination therapies in HCC were included. The severity and frequency of AEs were analyzed according to different treatment protocols.

**Results:**

A total of 58 studies (11 prospective, 47 retrospective) were included. HAIC monotherapy demonstrated relatively mild toxicity, primarily affecting hepatobiliary (transaminase elevation 53.2%, hypoalbuminemia 57.2%) and hematological systems (anemia 43.0%, thrombocytopenia 35.2%). HAIC with targeted therapy showed increased adverse events, including characteristic reactions like hand-foot syndrome (48.0%) and hypertension (49.9%). HAIC combined with targeted, and immunotherapy exhibited the highest adverse reaction rates (neutropenia 82.9%, transaminase elevation 97.1%), while HAIC with anti-angiogenic and immunotherapy showed a relatively favorable safety profile. Prospective studies consistently reported higher incidence rates than retrospective studies, suggesting potential underreporting in clinical practice.

**Conclusions:**

Different HAIC-based regimens exhibit distinct safety profiles requiring individualized management approaches. We propose a comprehensive framework for patient selection, monitoring strategies, and AE management. These recommendations aim to optimize treatment outcomes while minimizing adverse impacts on patient quality of life.

## 1 Introduction

Hepatocellular carcinoma (HCC) is one of the most common malignancies worldwide (1). In China, HCC not only maintains a high incidence rate but also shows a clear trend toward younger age groups, with new cases accounting for over 50% of the global total (2). Despite advances in diagnostic and therapeutic techniques, the prognosis remains poor (3, 4). While surgical resection represents the most effective curative treatment for HCC, only approximately 30% of patients are eligible for surgery, primarily due to advanced disease stage at diagnosis or insufficient liver function reserve (5). Hepatic arterial infusion chemotherapy (HAIC) enables high concentrations of chemotherapeutic agents in tumor tissues while reducing systemic exposure through arterial administration (6). This localized delivery strategy not only enhances local drug concentrations but also reduces systemic adverse reactions, making it an important option for treating unresectable HCC (7).

In recent years, with the continuous development of targeted therapy and immunotherapy, HAIC-based combination treatment strategies have become increasingly diverse (8). From HAIC monotherapy to HAIC combined with targeted therapy, and further to HAIC + targeted/anti-angiogenic + immunotherapy, the complexity and efficacy of treatment regimens have steadily improved (9–12). However, as combination therapy protocols expand, the spectrum of adverse reactions has changed significantly, presenting new safety challenges. In clinical practice, we have observed multi-system, multi-type adverse reactions, some of which may seriously affect patients’ treatment progress and prognosis. Currently, there is a lack of systematic review of the characteristics of adverse reactions and management strategies for HAIC and its combination therapies.

This review aims to summarize the characteristics of adverse reactions associated with HAIC and its various combination therapies through comprehensive analysis of existing research data and propose targeted management strategy recommendations. We hope that through this study, we can provide clinicians with more comprehensive guidance for adverse reaction management, thereby optimizing the safety of treatment protocols, developing individualized monitoring and prevention strategies, improving early recognition and management of adverse reactions, and ultimately optimizing treatment selection and adjustment to enhance patient benefits.

## 2 Methods

### 2.1 Literature search strategy

This study systematically searched relevant literature published in PubMed, Embase, Cochrane Library, and Web of Science databases from their inception until November 2024. The main search terms were: ((hepatic artery infusion chemotherapy [Title/Abstract]) OR (HAIC [Title/Abstract])) AND (hepatocellular carcinoma [Title/Abstract]). Additionally, to ensure search completeness, we supplemented with the strategy: ((“HAIC”) OR (“hepatic artery infusion”)) AND (“hepatocellular carcinoma” OR “liver cancer” OR “HCC”).

### 2.2 Literature selection

Inclusion criteria: (1) Clinical studies, including prospective and retrospective studies; (2) Study subjects were primary HCC patients; (3) Treatment protocols included HAIC monotherapy or combination therapy; (4) Complete adverse reaction data were reported; (5) Publications in English. Exclusion criteria: (1) Studies lacking adverse reaction data; (2) Duplicate publications; (3) Infusion protocols not based on oxaliplatin + 5-FU; (4) Sample size <5. For the systemic chemotherapy group, patients received FOLFOX4 regimen consisting of oxaliplatin 85 mg/m² administered intravenously (IV) on day 1, leucovorin (LV) 200 mg/m² IV infusion from hour 0 to 2 on days 1 and 2, followed by 5-fluorouracil (5-FU) 400 mg/m² IV bolus at hour 2, and then 5-FU 600 mg/m² as a 22-hour continuous IV infusion on days 1 and 2. This regimen was repeated every 2 weeks. For the HAIC group, treatment consisted of hepatic arterial infusion of oxaliplatin 85 mg/m², leucovorin 400 mg/m², and 5-fluorouracil 400 mg/m² as a bolus on day 1, followed by 5-fluorouracil 2400 mg/m² as a 24/46-hour continuous infusion. This regimen was administered every 3 weeks. All literature was independently screened by two researchers, with disagreements resolved through discussion.

### 2.3 Data extraction and organization

The following information was extracted from included studies: (1) Specific treatment protocols; (2) Incidence and grading of adverse reactions; (3) Management measures for adverse reactions. All adverse reactions were graded according to the National Cancer Institute Common Terminology Criteria for Adverse Events (CTCAE), categorized as mild-to-moderate (Grade I-II) and severe (Grade III-IV).

### 2.4 Data analysis

Studies were classified into four categories based on treatment protocols: HAIC monotherapy, HAIC + targeted therapy, HAIC + targeted + immunotherapy, and HAIC + anti-angiogenic + immunotherapy. Adverse reactions were categorized by organ systems, including hematological (leukopenia, neutropenia, thrombocytopenia, anemia), hepatobiliary (transaminase elevation, bilirubin elevation, hypoalbuminemia), gastrointestinal (nausea, vomiting, diarrhea, abdominal pain), cardiovascular (hypertension), dermatological (hand-foot syndrome, rash), neurological (sensory neuropathy), and immune-related adverse reactions (RCCEP, hypothyroidism, immune hepatitis, etc.).

### 2.5 Statistical methods

Descriptive statistical methods were used, with adverse reaction rates expressed as median and range (minimum-maximum). The top 20 high-incidence adverse reactions were analyzed in detail, with stratified analysis by system classification and severity. Analysis focused on: (1) Main adverse reaction spectrum of each treatment protocol; (2) Identification of high-incidence adverse reactions; (3) Characteristics of severe adverse reactions; (4) Newly emerging characteristic adverse reactions; (5) Toxicity accumulation effects of combination therapy. R software (R 4.2.2) was used for visualization.

## 3 Results

### 3.1 Overall characteristics of adverse reactions

This study included 58 studies, comprising 11 prospective clinical (9–11, 13–20) studies and 47 retrospective clinical studies (12, 21–67). Adverse reactions primarily involved hematological, hepatobiliary, gastrointestinal, cardiovascular, dermatological, neurological systems, and immune-related reactions. Overall, hematological and hepatobiliary adverse reactions were most common, followed by gastrointestinal reactions. As treatment protocols became more complex, the spectrum of adverse reactions gradually expanded, with corresponding increases in characteristic adverse reactions.

### 3.2 Comparison between prospective and retrospective studies

Prospective clinical studies (11) and retrospective studies (47) showed consistency in adverse reaction distribution patterns, with similar rankings of major adverse reactions. In HAIC monotherapy, both types of studies showed hepatobiliary (transaminase elevation, hypoalbuminemia) and hematological (anemia, thrombocytopenia) adverse reactions as most common; in combination therapy protocols, both demonstrated characteristic adverse reactions (such as targeted therapy-related hand-foot syndrome, immunotherapy-related RCCEP). However, there were significant differences in adverse reaction incidence rates between the two types of studies.

**Figure 1:**
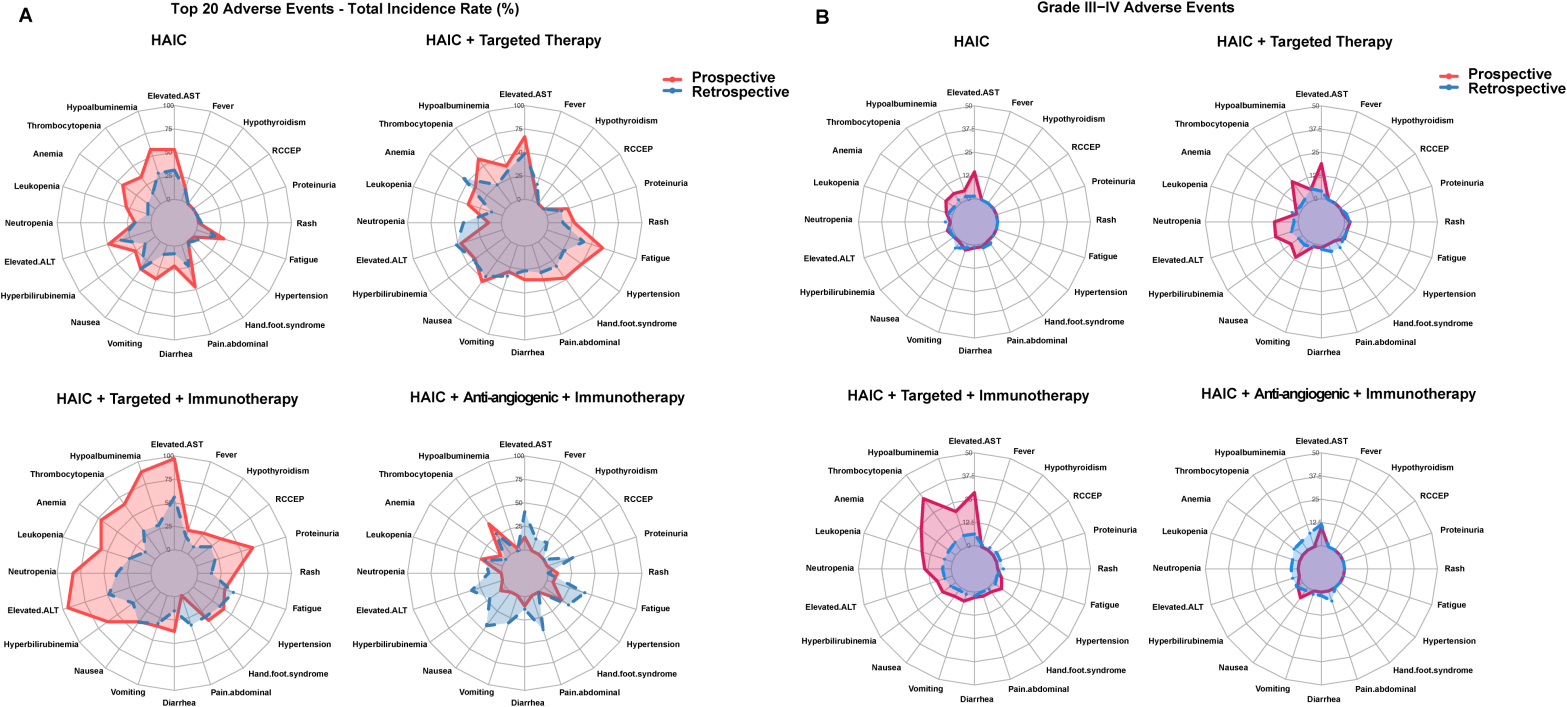
Comparison of adverse reaction rates between prospective and retrospective studies. **(A)** Radar chart comparing overall adverse reaction rates; **(B)** Radar chart comparing Grade III-IV adverse reaction rates.

Prospective studies generally reported higher incidence rates of adverse reactions compared to retrospective studies. Taking HAIC + targeted + immunotherapy as an example, neutropenia (82.9% vs 36.1%), thrombocytopenia (65.7% vs 30.8%), and transaminase elevation (97.1% vs 56.1%) all showed significant differences (**Figure 1A**). Differences in Grade III-IV adverse reactions were equally notable, such as neutropenia (34.3% vs 5.2%) and transaminase elevation (28.6% vs 6.3%) (**Figure 1B**). This difference in incidence rates, while maintaining relatively consistent distribution characteristics of adverse reactions, suggests that prospective studies may have obtained more complete safety data.

### 3.3 Analysis of adverse reaction characteristics in different treatment protocols

Different treatment protocols showed unique distribution characteristics of adverse reactions through radar chart analysis, with the spectrum of adverse reactions gradually expanding and severity increasing from HAIC monotherapy to multi-drug combination therapy. Given that adverse reactions are more comprehensively documented in prospective clinical trials, we conducted comparative analyses of adverse events between HAIC-based regimens and various standard treatments including systemic chemotherapy (FOLFOX4), targeted therapy, targeted therapy + immunotherapy, and anti-angiogenic therapy + immunotherapy.

#### 3.3.1 HAIC monotherapy

**Figure 2:**
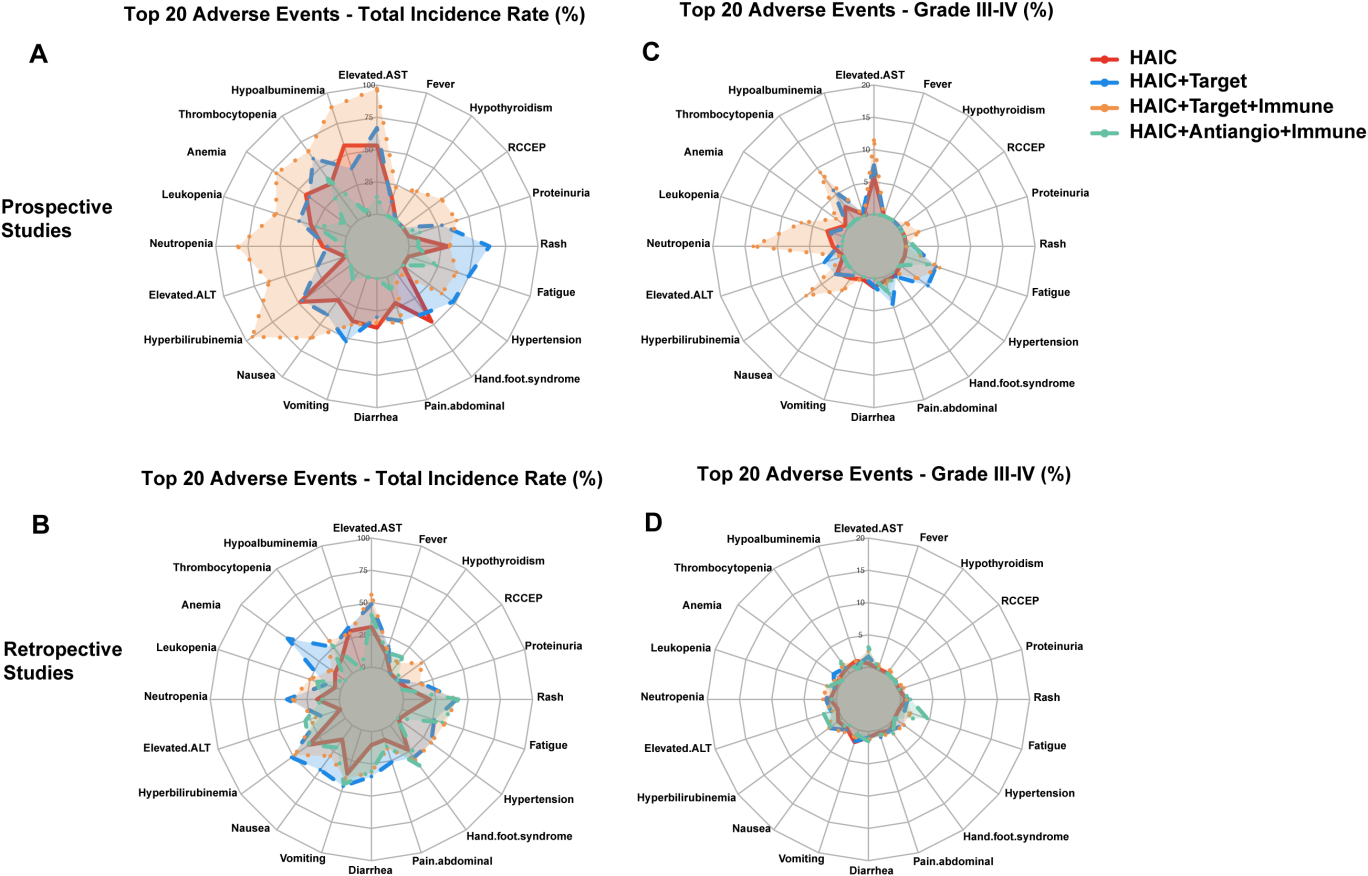
Comparison of adverse reaction rates across HAIC and its combination therapies. **(A)** Overall incidence rates - prospective studies **(B)** Overall incidence rates - retrospective studies. **(C)** Grade III-IV adverse reaction rates - prospective studies **(D)** Grade III-IV adverse reaction rates - retrospective studies.

HAIC monotherapy demonstrated a relatively mild adverse reaction spectrum. Adverse reactions primarily involved hepatobiliary and hematological systems, showing a “dual-peak” distribution. Prospective studies revealed that hepatobiliary manifestations mainly included transaminase elevation (53.2%) and hypoalbuminemia (57.2%), while hematological manifestations primarily included anemia (43.0%) and thrombocytopenia (35.2%) (**Figure 2A**). Although retrospective studies showed lower incidence rates, the distribution characteristics were similar, with transaminase elevation (31.1%) and thrombocytopenia (14.1%) remaining the primary manifestations. Gastrointestinal reactions such as nausea (35.9%), vomiting (38.0%), and abdominal pain (47.0%), although not infrequent, were almost entirely mild to moderate, with good overall patient tolerability (**Figure 2B**). Grade III-IV adverse reactions showed a “low-level dispersed” distribution, with only transaminase elevation reaching 14.5%, while others remained below 10% (**Figures 2C, D**).

**Figure 3:**
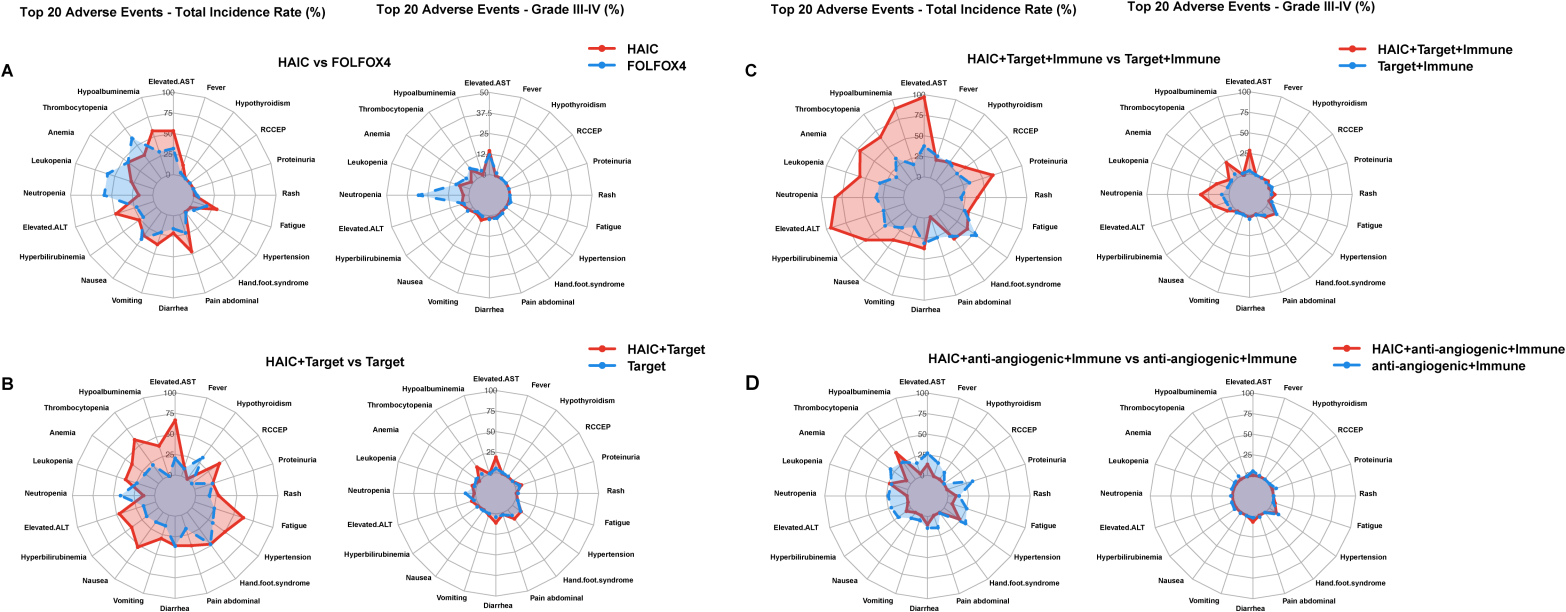
Comparison of adverse reaction rates across HAIC and its other therapies. **(A)** Overall and Grade III-IV Adverse Events: HAIC versus FOLFOX4 in prospective studies; **(B)** Overall and Grade III-IV Adverse Events: HAIC + targeted versus targeted in prospective studies; **(C)** Overall and Grade III-IV Adverse Events: HAIC + targeted + immunotherapy versus targeted + immunotherapy in prospective studies; **(D)** Overall and Grade III-IV Adverse Events: HAIC + anti-angiogenic + immunotherapy versus anti-angiogenic + immunotherapy in prospective studies.

Compared to intravenous chemotherapy, HAIC demonstrated a milder adverse reaction profile with better tolerability (68, 69). While intravenous chemotherapy (FOLFOX4) showed higher incidences of hematological toxicity, including neutropenia (59.02%) and thrombocytopenia (60.66%), HAIC primarily involved hepatobiliary toxicity, such as transaminase elevation (53.2%) and hypoalbuminemia (57.2%). Notably, HAIC had significantly fewer Grade III-IV adverse reactions, with transaminase elevation at 14.5% as the most common, while severe hematological toxicities were rare (e.g., neutropenia 3.4%). In contrast, FOLFOX4 demonstrated a markedly higher rate of severe adverse reactions, particularly neutropenia (30.6%) and thrombocytopenia (7.65%), highlighting its stronger bone marrow suppressive effects (**Figure 3A**).

#### 3.3.2 HAIC combined with targeted therapy

When HAIC was combined with targeted agents, both prospective and retrospective study data showed significantly expanded adverse reaction spectra, presenting a “multi-system balanced” distribution (**Figures 2A, B**). First, the incidence of thrombocytopenia increased significantly to 59.0%, with Grade III-IV reaching 14.3%; transaminase elevation increased to 66.7%, with Grade III-IV at 18.9%. Additionally, characteristic adverse reactions specific to targeted therapy emerged: hand-foot syndrome (48.0%, with Grade III-IV at 13.4%) and hypertension (49.9%, Grade III-IV at 12.8%) became issues requiring special attention. The incidence of fatigue (62.2%) also increased significantly compared to monotherapy (**Figures 2C, D**). Although overall adverse reaction rates increased, most remained controllable Grade I-II reactions.

Comparative analysis between HAIC + targeted therapy versus targeted therapy alone demonstrated distinct safety patterns (70–77). The HAIC combination showed higher incidences in several adverse events, particularly in hepatic dysfunction (elevated transaminases: 66.7% vs 20.35%, grade III-IV: 18.9% vs 5.3%) and certain hematological toxicities, especially thrombocytopenia (59.0% vs 21.1%, grade III-IV: 14.3% vs 4.1%). Interestingly, neutropenia was less frequent in the HAIC combination group (13.5% vs 41.5%, grade III-IV: 1.5% vs 12.0%). Gastrointestinal reactions showed varied patterns, with notably higher rates of nausea (52.5% vs 14.7%) but similar rates of diarrhea (35.8% vs 36.5%). The HAIC combination also resulted in increased incidences of fatigue (62.2% vs 25.0%) and hypertension (49.9% vs 31.5%). Although most adverse events remained grade I-II, these findings suggest that while the combination strategy may potentially offer therapeutic benefits, it requires careful monitoring, especially for hepatic function and platelet counts (**Figure 3B**).

#### 3.3.3 HAIC combined with targeted and immunotherapy

HAIC + targeted + immunotherapy demonstrated the most complex adverse reaction characteristics, showing an “overall elevation” pattern (**Figures 2A, B**). The most significant changes were substantial increases in hematological toxicity: neutropenia reached 82.9%, leukopenia 57.1%, and anemia 71.4%. Liver function abnormalities reached peak levels, with transaminase and ALT elevations reaching 97.1% and 94.3%, respectively. Meanwhile, immune-related adverse reactions emerged as new challenges, including RCCEP (37.1%), hypothyroidism (27.8%), and immune-related dermatitis (16.7%). Furthermore, regarding more serious complications such as gastrointestinal bleeding, the incidence increased from 1.9% in the HAIC monotherapy group to 7.7% in the HAIC + targeted + immunotherapy group, with a notable increase in Grade III-IV bleeding (3.6%) (**Figures 2C, D**). This difference may be related to the additional effects of immunotherapy and anti-angiogenic therapy on gastrointestinal mucosa.

Grade III-IV adverse reactions showed a “prominent peak” pattern, with neutropenia (34.3%) and transaminase elevation (28.6%) showing markedly increased incidence rates. Although overall adverse reaction rates were highest, most were Grade I-II reactions, with relatively controllable proportions of Grade III-IV reactions.

Comparative analysis between HAIC + targeted therapy + immunotherapy versus targeted therapy + immunotherapy demonstrated notably increased adverse events (78–86). The HAIC combination showed significantly higher incidences of hepatic dysfunction (elevated transaminases: 97.1% vs 37.5%, grade III-IV: 28.6% vs 4.15%) and hematological toxicities, particularly in neutropenia (82.9% vs 33.3%, grade III-IV: 34.3% vs 8.75%), leukopenia (57.1% vs 31.55%, grade III-IV: 17.1% vs 3.1%), and thrombocytopenia (65.7% vs 33.55%, grade III-IV: 22.9% vs 5.8%). The combination also led to increased rates of hypoalbuminemia (88.6% vs 17.3%). Gastrointestinal reactions showed a similar pattern of elevation, with higher rates of nausea (38.9% vs 20.5%) and vomiting (34.3% vs 13.0%). While most adverse events remained at grade I-II, the substantial increase in grade III-IV events, particularly in hematological and hepatic parameters, suggests the need for more intensive monitoring and management strategies (**Figure 3C**).

#### 3.3.4 HAIC combined with anti-angiogenic and immunotherapy

Limited data is available for this combination therapy. Compared to other combination regimens, this showed milder toxicity characteristics, presenting a “relatively concentrated” pattern. Overall adverse reaction rates were significantly lower compared to other combination protocols (**Figures 2A, B**). Hematological toxicity was relatively mild, with leukopenia at only 23.3% and no Grade III-IV adverse reactions; liver function-related adverse reactions also decreased significantly, with transaminase elevation at only 13.3%. Special attention was required for hypertension (23.3%, Grade III-IV 10%) and proteinuria (28.8%). Gastrointestinal reactions were generally controllable, with diarrhea occurring in 10% of cases, including 6.7% Grade III-IV (**Figures 2C, D**). However, current data on this aspect is limited, possibly due to economic factors.

In contrast to other combination approaches, HAIC + anti-angiogenic + immunotherapy demonstrated unique safety characteristics (87–92). Unexpectedly, this combination showed lower incidence rates of adverse events in several aspects compared to anti-angiogenic + immunotherapy. Notably, reduced frequencies were observed in hepatic dysfunction (elevated transaminases: 13.3% vs 26.9%), anemia (6.7% vs 30.55%), and fatigue (6.7% vs 25.2%), suggesting better tolerability. The only markedly increased adverse event was thrombocytopenia (40.0% vs 25.8%). Furthermore, grade III-IV adverse events were relatively infrequent, with hypertension being the primary concern (10.0%). These findings suggest that HAIC plus anti-angiogenic and immunotherapy might represent a relatively well-tolerated treatment option, although careful monitoring of thrombocytopenia and hypertension remains essential (**Figure 3D**).

### 3.4 Individualized treatment selection and adverse reaction management strategies

Based on the analysis of adverse reaction characteristics of HAIC and its combination therapies, we need to establish systematic management strategies, from patient selection to continuous monitoring, to ensure treatment safety and efficacy.

#### 3.4.1 Principles for individualized treatment protocol selection

Patient baseline status is a key consideration when selecting treatment protocols. For patients with good liver function reserve and no significant underlying diseases, HAIC + targeted + immunotherapy may provide maximum benefit. However, this protocol has higher adverse reaction rates (neutropenia 82.9%, transaminase elevation 97.1%), and the increased risk of gastrointestinal bleeding with HAIC + targeted + immunotherapy requires caution in patients with high-risk factors for gastrointestinal bleeding. Nevertheless, these adverse reactions are mostly controllable through standardized management. For patients with certain underlying diseases or moderate liver function reserve, HAIC combined with targeted therapy may be more suitable, with primary focus on specific adverse reactions such as hand-foot syndrome (48.0%) and hypertension (49.9%). For high-risk patients (poor liver function reserve or significant underlying diseases), HAIC monotherapy’s mild adverse reaction profile (transaminase elevation 53.2%, thrombocytopenia 35.2%) makes it a better choice.

Further research on the mechanisms of adverse reactions and their prevention is needed. For instance, a retrospective study showed that 64.6% of patients experienced abdominal pain during HAIC, possibly due to vascular spasm caused by oxalate (a degradation product of oxaliplatin) irritating blood vessels, or insufficient hepatic blood supply due to small vessel diameter (32). Effective pain management and the use of lidocaine for antispasmodic effects during infusion can effectively relieve abdominal pain during treatment.

#### 3.4.2 Stratified management strategies

**Figure 4:**
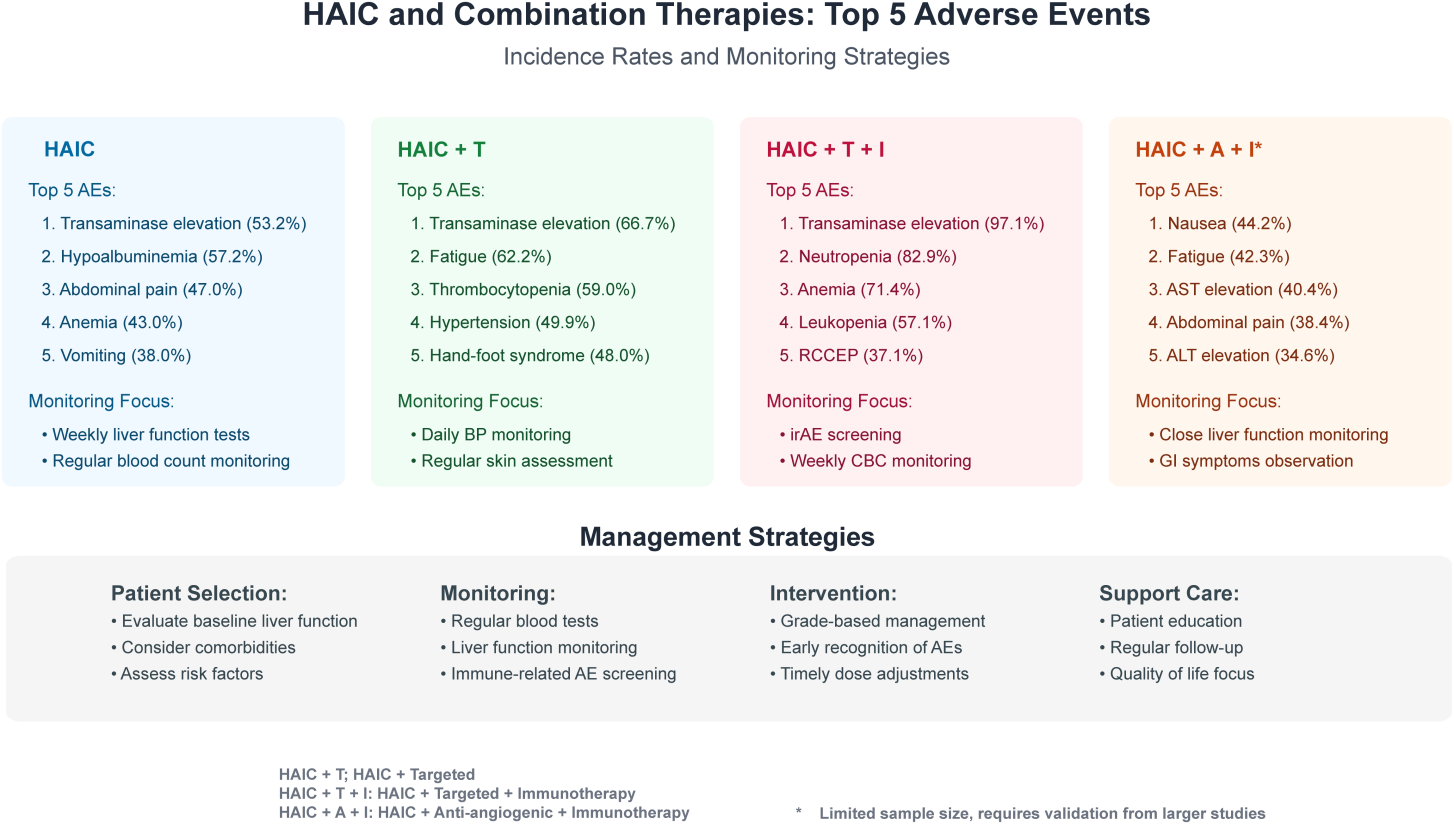
Management strategies for adverse reactions in HAIC and HAIC-based combination therapies.

Management of adverse reactions should be based on severity level. Grade I-II adverse reactions usually allow continued treatment with symptomatic support; Grade III reactions require considering treatment suspension or dose adjustment, with gradual resumption after improvement; Grade IV reactions require immediate drug discontinuation and active treatment. Specific monitoring plans should be developed for characteristic adverse reactions of different treatment protocols (**Figure 4**):

- HAIC: Focus on monitoring complete blood count and liver function
- HAIC + targeted therapy: Enhanced monitoring of hand-foot syndrome and blood pressure
- HAIC + targeted + immunotherapy: Comprehensive monitoring plan, especially for immune-related adverse reactions and gastrointestinal bleeding
- HAIC + anti-angiogenic + immunotherapy: Focus on blood pressure and proteinuria

#### 3.4.3 Establishment of long-term management systems

Successful treatment requires a comprehensive long-term management system. First, establish standardized follow-up protocols, including regular efficacy assessment and adverse reaction monitoring. Second, enhance patient education to improve awareness of early adverse reaction symptoms, promoting early detection and management. Finally, maintain good physician-patient communication to ensure timely handling of problems.

This stratified management strategy should be based on standardized monitoring. Set appropriate monitoring items and frequencies according to different treatment protocols, including routine hematological examinations, biochemical indicator monitoring, and screening for specific adverse reactions. Meanwhile, regularly evaluate treatment effectiveness and adjust treatment protocols based on patient tolerance and response.

Overall, management of HAIC and its combination therapies should be an individualized and dynamically adjusted process. Through reasonable protocol selection, systematic monitoring systems, and timely intervention measures, adverse reactions’ impact can be minimized while ensuring treatment effectiveness and improving patient quality of life. Importantly, the higher adverse reaction rates shown in prospective studies suggest that we may need closer monitoring in actual clinical work to detect and address potential problems promptly.

## 4 Future perspectives

As HAIC and its combination therapy protocols become widely used in HCC treatment, there remains room for further optimization in understanding and managing adverse reactions. The main limitation of current research lies in the significant data discrepancy between prospective and retrospective studies, suggesting potential inadequacies in adverse reaction monitoring and reporting in actual clinical work. For example, the reported incidence of leukopenia in prospective studies (28.8%) is much higher than in retrospective studies (4.7%), indicating the need for more standardized adverse reaction monitoring and reporting systems.

Future research should focus on several aspects: First, more high-quality prospective studies are needed to validate the safety characteristics of different combination protocols. Particularly for new combination protocols such as HAIC + anti-angiogenic + immunotherapy, their relatively low adverse reaction rates require more data support. Second, research on predictive factors and early identification indicators for immune-related adverse reactions is also important, which will help improve the safety of immunotherapy-containing protocols such as HAIC + targeted + immunotherapy. Finally, establishing standardized adverse reaction assessment systems to promote multi-center data comparability and reliability is crucial.

Regarding optimization of management strategies, there is a need to explore more individualized treatment selection criteria and establish prediction models based on patient characteristics for more accurate assessment of adverse reaction risks. Meanwhile, with the development of telemedicine technology, consideration should be given to establishing more convenient adverse reaction monitoring and follow-up systems to improve management efficiency.

## 5 Conclusion

Through systematic analysis of adverse reaction data from HAIC and its combination therapy protocols, this review finds that different treatment combinations have their unique safety characteristics. HAIC monotherapy shows a relatively mild adverse reaction spectrum, mainly manifesting as controllable hematological toxicity and liver function abnormalities. HAIC combined with targeted therapy adds specific reactions such as hand-foot syndrome and hypertension to the basic adverse reactions. Although HAIC + targeted + immunotherapy has the highest adverse reaction rates, most are controllable Grade I-II reactions. HAIC combined with anti-angiogenic and immunotherapy shows relatively favorable safety characteristics, providing a new option for specific patient populations.

The higher adverse reaction rates generally reported in prospective studies, compared to retrospective studies, more closely reflect real clinical situations, suggesting the need for more cautious and standardized monitoring strategies in actual practice. Based on these findings, we recommend individualized treatment selection and stratified management strategies, including protocol selection based on patient characteristics, systematic monitoring plans, and timely intervention measures.

For clinical practice, we recommend: First, strictly evaluate patient baseline status to select appropriate treatment protocols; second, establish comprehensive monitoring systems for early detection and timely intervention; finally, maintain regular follow-up and dynamically adjust treatment strategies. Only through such systematic management can we ensure treatment effectiveness while maximizing control of adverse reactions’ impact and improving patient benefits.

In the future, with the accumulation of more high-quality research data and optimization of management strategies, the application of HAIC and its combination therapies in HCC treatment will become more standardized and individualized, providing safer and more effective treatment options for patients.

Several limitations of this study should be acknowledged. First, although we conducted a comprehensive review of adverse events across different treatment protocols, some relevant literature might have been missed despite our best efforts to minimize selection bias. Second, the heterogeneous nature of the source data, particularly the limited number of studies for certain combination therapies, may have introduced statistical bias in our comparative analyses. Third, baseline characteristics of patients varied across different studies, potentially confounding the comparison of adverse event profiles. Fourth, the safety data for some combination therapies, especially HAIC plus anti-angiogenic and immunotherapy, remains limited and requires further validation through larger, prospective clinical trials. These limitations underscore the need for more standardized, prospective studies with uniform adverse event reporting criteria to better evaluate the safety profiles of various HAIC-based combination therapies.
